# Designs and Methodologies Used in Landmark Clinical Trials of Glaucoma: Implications for Future Big Data Mining and Actionable Disease Treatment

**DOI:** 10.3389/fmed.2022.818568

**Published:** 2022-01-26

**Authors:** Saif Aldeen AlRyalat, Monica K. Ertel, Leonard K. Seibold, Malik Y. Kahook

**Affiliations:** ^1^Department of Ophthalmology, The University of Jordan, Amman, Jordan; ^2^Department of Ophthalmology, Sue Anschutz-Rodgers Eye Center, University of Colorado School of Medicine, Aurora, CO, United States

**Keywords:** glaucoma, open angle, clinical trials, diagnosis, optic nerve

## Introduction

A widely agreed upon definition of glaucoma with clear diagnostic criteria to classify disease presence and status remains elusive in both clinical and research settings. For decades, the diagnosis of glaucoma was primarily based on documenting visual field changes through static or kinetic perimetry and correlating these findings with structural changes at the optic nerve head (1, 2). Recently, the 10th World Glaucoma Association (WGA) Consensus Meeting supported the use of optic nerve structural endpoints alone to provide sufficient information for a diagnosis of glaucoma, even in the absence of visual field changes, a state termed pre-perimetric glaucoma (3). The National Institute for Health and Care Excellence (NICE), the national body tasked with issuing clinical guidelines for England and Wales, does not provide strict criteria to diagnose glaucoma (4). There is scarce guidance on criteria to definitively diagnose glaucoma which leads to difficulties in research efforts focused on phenotyping ocular imaging/diagnostic data as a first step toward identifying and predicting disease to enhance clinical care.

## Landmark Trials in Glaucoma

The field of glaucoma has the second largest number of published randomized controlled trials in all of ophthalmology, the majority of which evaluate glaucoma treatments (5, 6). Landmark randomized controlled trials have shaped the practice of glaucoma care and are commonly used to teach medical learners the basics of treating patients with suspected or diagnosed glaucomatous optic neuropathy (7–10). While it is clear that these trials have provided a wealth of information for practical patient care, the added benefit of these data sets is the ability to mine information that can guide the planning of future studies. This can take the form of feeding large labeled data sets into novel machine learning algorithms which potentially can produce new findings to inform patient care (11, 12). The large data sets from previous landmark trials are often published in top tier journals and receive a great deal of attention. However, the building blocks of these studies; including methodologies, patient selection criteria and diagnostic operating procedures, are usually published in a separate document and receive less rigorous attention (1, 2, 13). The aim of this brief report is to clarify the diagnostic criteria used by landmark glaucoma trials with a focus on design and methodology.

We reviewed diagnostic criteria used by landmark glaucoma clinical trials commonly cited in ophthalmic textbooks and review articles (7–10). We also supplemented the mentioned trials by an advanced PubMed search for design and methodology articles published for glaucoma clinical trials where we used the following search strategy:

((Design[Title]) OR (Method[Title])) AND (Glaucoma[Title])

We included trials that were concerned mainly with open angle glaucoma, as this represents the majority of the effort in this space. Studies that had design protocols published in a separate “design and methodology” article were of particular interest.

**Figure 1:**
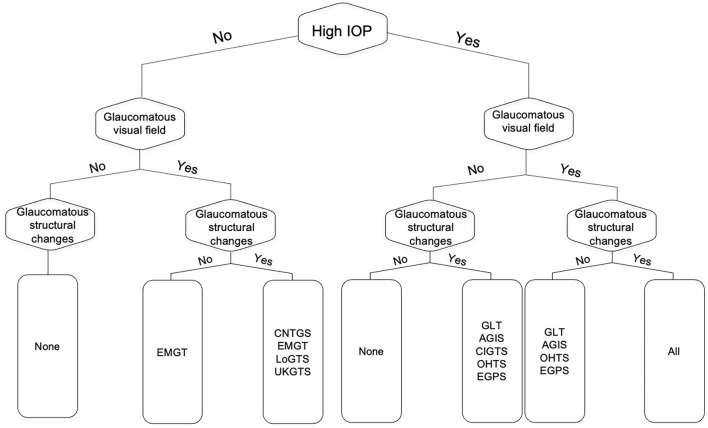
Flow chart of the different combinations from the three glaucoma diagnostic facets (functional, structural, and intraocular pressure) as they relate to the major clinical trials in glaucoma. GLT, The Glaucoma Laser Trial; AGIS, The Advanced Glaucoma Intervention Study; CNTGS, Collaborative Normal-Tension Glaucoma Study; OHTS, Ocular Hypertension Treatment Study; EMGT, Early Manifest Glaucoma Trial; EGPS, The European Glaucoma Prevention Study; LoGTS, Low-Pressure Glaucoma Treatment Study; UKGTS, UK Glaucoma Treatment Study.

Fourteen trials were identified in our assessment, however, five of them were surgical trials focused on outcome comparisons and used criteria previously utilized in larger randomized clinical trials (14–18). As a result, we focused on nine clinical trials (1, 2, 13, 19–24). Glaucoma diagnosis was based on one or more of the following criteria: functional criteria in terms of visual field performance, structural criteria in terms of optic disc features, and/or intraocular pressure (IOP). Table 1 details the specific glaucoma diagnostic criteria adopted by each of the landmark clinical trials. Visual field criterion was a pre-requisite diagnostic criterion for most clinical trials, although they varied in their definition for what qualified as a glaucomatous visual field (1, 13, 19, 22, 23, 25, 26). The Glaucoma Laser Trial (GLT) and the Collaborative Initial Glaucoma Treatment Study (CIGTS) did not require visual fields for patients with an IOP of 27 or higher, where only structural evidence of glaucomatous optic disc damage was required (1, 13). More recent trials, including the European Glaucoma Prevention Study (EGPS) (22), Low-Pressure Glaucoma Treatment Study (LoGTS) (23), and UK Glaucoma Treatment Study (UKGTS) (18), required structural glaucomatous features and/or visual field glaucomatous features. Figure 1 shows how the different combinations of functional, structural and IOP criteria were utilized across these studies.

**Table 1:** Landmark glaucoma clinical trials and each of the functional, structural, and intraocular pressure criteria used in glaucoma diagnosis or enrollment.

**Trial**	**Year**	**IOP (mmHg) criteria**	**Visual field criteria**	**Structural criteria**
The Glaucoma Laser Trial (GLT) (1)	1991	>21	Glaucomatous visual field defect/deterioration on Program 32 (1)	None
		≥27	-	cup/disc ratio disparity ≥0.3
		≥31	-	cup/disc ratio ≥0.8
The Advanced Glaucoma Intervention Study (AGIS) (2)	1994	>21	Visual field defect score of at least (25)	-
		17–21	Visual field deterioration	Disc rim deterioration
Collaborative Normal-Tension Glaucoma Study (CNTGS) (19)	1998	≤24^*^	Glaucomatous visual field defect/deterioration on Program 32 (19)	Glaucomatous disc judged by physicians
The Collaborative Initial Glaucoma Treatment Study (CIGTS) (13)	1999	≥20	At least three contiguous points on the total deviation probability plot at the <2% level and a Glaucoma Hemifield Test result that is “outside normal limits,” (25)	Glaucomatous optic disc
		20–26	At least two contiguous points in the same hemifield on the total deviation probability plot at the <2% level (25)	Glaucomatous optic disc
		≥27	-	Glaucomatous optic disc
Ocular Hypertension Treatment Study (OHTS) (20)	1999	≥24 and ≤32^*^	Corrected pattern standard deviation <0.05 OR glaucoma hemifield test outside normal limits (26)	-
			-	Stereoscopic optic disc photographs showing a change in the position of vessels (greater than expected by eye movement), development of notch, pit, or development of thinning or pallor in the neural rim.
Early Manifest Glaucoma Trial (EMGT) (21)	1999	<30^*^	Glaucoma hemifield test outside normal limits (26)	-
			Glaucoma hemifield test borderline (26)	Glaucomatous optic disc features correspond to visual field
The European Glaucoma Prevention Study (EGPS) (22)	2002	>21 to ≤29^*^	Deterioration from baseline (22)	-
			-	Deterioration from baseline
Low-Pressure Glaucoma Treatment Study (LoGTS) (23)	2005	≤21^*^	At least 3 contiguous points depressed more than 8 decibels or 2 contiguous points depressed more than 10 decibels (23)	Glaucomatous optic disc consistent with visual field
UK Glaucoma Treatment Study (UKGTS) (18)	2013	<30^*^	Reduction in sensitivity at 2 or more contiguous points with *P* <0.01 loss or more, 3 or more contiguous points with *P* <0.05 loss or more, or a 10-dB difference across the nasal horizontal midline at 2 or more adjacent points in the total deviation plot (27).	Cup-to-disc ratio of ≥0.7, focal narrowing of the neural rim, or both

* *These intraocular pressure thresholds were used for enrollment, rather than for establishing the diagnosis*.

## Discussion

Detailing the presence and/or progression of glaucomatous optic neuropathy is based on the functional and structural characteristics of the optic nerve, relying on the combination of both subjective and objective data obtained from clinical examination and output from various diagnostic modalities. While large randomized clinical trials have provided insights into the treatment of glaucoma, the criteria used for enrollment and interventions in each trial are disparate, making application of findings in the clinical setting difficult at best. With the emergence of artificial intelligence and machine learning techniques that seek to decipher new learnings from these large datasets, a deep and nuanced understanding of the designs and methodologies used is key to unlocking even more data to enhance patient care. We have provided an overview of diagnostic criteria used in landmark randomized controlled trials of open angle glaucoma. These criteria differ in the diagnostic weight placed on subjective visual field studies, objective structural changes of the optic nerve, as well as the use of IOP metrics. Outlining these criteria in a single resource may act as a starting point for discussions on proper methods of mining past data sets while also reaching some consensus for implementing a commonly agreed upon set of diagnostic criteria in future studies to facilitate broader analyses. The ultimate goal is to make findings from large randomized clinical trials more actionable in a real-world clinical setting by leveraging big data sets toward predictive output and guidance for when to observe and when to intervene with escalating care.

## Author Contributions

SA, ME, LS, and MK contributed in research conception, literature review, and manuscript writing. All authors contributed to the article and approved the submitted version.

## Conflict of Interest

The authors declare that the research was conducted in the absence of any commercial or financial relationships that could be construed as a potential conflict of interest.

## Publisher's Note

All claims expressed in this article are solely those of the authors and do not necessarily represent those of their affiliated organizations, or those of the publisher, the editors and the reviewers. Any product that may be evaluated in this article, or claim that may be made by its manufacturer, is not guaranteed or endorsed by the publisher.

## References

[1] The Glaucoma Laser Trial (GLT): 3. Design and methods. Glaucoma laser trial research group. Control Clin Trials. (1991) 12:504–24. 10.1016/0197-2456(91)90010-J 1657527

[2] Ederer F Gaasterland DE Sullivan EK. The Advanced Glaucoma Intervention Study (AGIS): 1. Study design and methods and baseline characteristics of study patients. Control Clin Trials. (1994) 15:299–325. 10.1016/0197-2456(94)90046-9 7956270

[3] Weinreb RN Garway-Heath DF Leung C Medeiros FA Liebmann J. Diagnosis of Primary Open Angle Glaucoma: WGA consensus series - 10. Kugler Publications (2017).

[4] Overview | Glaucoma: diagnosis management | Guidance | NICE. Available online at: https://www.nice.org.uk/guidance/ng81 (accessed April 2, 2021).

[5] AlRyalat SA Abukahel A Elubous KA. Randomized controlled trials in ophthalmology: a bibliometric study. F1000Res. (2019) 8:1718. 10.12688/f1000research.20673.1 32318264 PMC7156022

[6] Storgaard L Tran TL Freiberg JC Hauser AS Kolko M. Glaucoma clinical research: trends in treatment strategies and drug development. Front Med. (2021) 8:733080. 10.3389/fmed.2021.733080 34589504 PMC8473801

[7] Penman A Crowder K Watkins WM. 50 Studies Every Ophthalmologist Should Know. Oxford University Press (2020). 10.1093/med/9780190050726.001.0001

[8] Wishart PK. Interpretation of the glaucoma “landmark studies.” *Br J Ophthalmol*. (2009) 93:561–2. 10.1136/bjo.2008.145532 19395626

[9] Henein C Mathew RG. Use of infographics to communicate landmark glaucoma trials. Eye. (2021) 35:2073–4. 10.1038/s41433-020-01369-x 33469133 PMC8302723

[10] Dohlman JC Lorch AC. Pivotal Trials in Ophthalmology: A Guide for Trainees. Springer Nature (2021). 10.1007/978-3-030-63978-5

[11] Medeiros FA Jammal AA Thompson AC. From machine to machine: An OCT-trained deep learning algorithm for objective quantification of glaucomatous damage in fundus photographs. Ophthalmology. (2019) 126:513–21. 10.1016/j.ophtha.2018.12.033 30578810 PMC6884092

[12] Thompson AC Jammal AA Medeiros FA. A review of deep learning for screening, diagnosis, and detection of glaucoma progression. Trans Vis Sci Tech. (2020) 9:42. 10.1167/tvst.9.2.42 32855846 PMC7424906

[13] Musch DC Lichter PR Guire KE Standardi CL. The Collaborative Initial Glaucoma Treatment Study: study design, methods, and baseline characteristics of enrolled patients. Ophthalmology. (1999) 106:653–62. 10.1016/S0161-6420(99)90147-1 10201583

[14] Gedde SJ Schiffman JC Feuer WJ Parrish RK Heuer DK Brandt JD. The tube versus trabeculectomy study: design and baseline characteristics of study patients. Am J Ophthalmol. (2005) 140:275–87. 10.1016/j.ajo.2005.03.031 16086949

[15] Christakis PG Tsai JC Zurakowski D Kalenak JW Cantor LB Ahmed IIK. The ahmed versus baerveldt study: design, baseline patient characteristics, and intraoperative complications. Ophthalmology. (2011) 118:2172–9. 10.1016/j.ophtha.2011.05.003 21906813

[16] Gedde SJ Chen PP Heuer DK Singh K Wright MM Feuer WJ. Methodology of a multicenter randomized clinical trial comparing tube shunt surgery trabeculectomy with mitomycin C. Ophthalmology. (2018) 125:774–81. 10.1016/j.ophtha.2017.10.037 29248173 PMC9289720

[17] Gazzard G Konstantakopoulou E Garway-Heath D Barton K Wormald R Morris S. Laser in Glaucoma and Ocular Hypertension (LiGHT) trial. A multicentre, randomised controlled trial: design and methodology. Br J Ophthalmol. (2018) 102:593–8. 10.1136/bjophthalmol-2017-310877 28903966

[18] King AJ Fernie G Azuara-Blanco A Burr JM Garway-Heath T Sparrow JM. Treatment of Advanced Glaucoma Study: a multicentre randomised controlled trial comparing primary medical treatment with primary trabeculectomy for people with newly diagnosed advanced glaucoma-study protocol. Br J Ophthalmol. (2018) 102:922–8. 10.1136/bjophthalmol-2017-310902 29074496 PMC6047148

[19] Comparison of glaucomatous progression between untreated patients with normal-tension glaucoma and patients with therapeutically reduced intraocular pressures. Collaborative Normal-Tension Glaucoma Study Group. Am J Ophthalmol. (1998) 126:487–97. 10.1016/S0002-9394(98)00223-2 9780093

[20] Gordon MO Kass MA. The Ocular Hypertension Treatment Study: design and baseline description of the participants. Arch Ophthalmol. (1999) 117:573–83. 10.1001/archopht.117.5.573 10326953

[21] Leske MC Heijl A Hyman L Bengtsson B. Early Manifest Glaucoma Trial: design and baseline data. Ophthalmology. (1999) 106:2144–53. 10.1016/S0161-6420(99)90497-9 10571351

[22] Miglior S Zeyen T Pfeiffer N Cunha-Vaz J Torri V Adamsons I. The European glaucoma prevention study design and baseline description of the participants. Ophthalmology. (2002) 109:1612–21. 10.1016/S0161-6420(02)01167-3 12208707

[23] Krupin T Liebmann JM Greenfield DS Rosenberg LF Ritch R Yang JW. The Low-pressure Glaucoma Treatment Study (LoGTS) study design and baseline characteristics of enrolled patients. Ophthalmology. (2005) 112:376–85. 10.1016/j.ophtha.2004.10.034 15745762

[24] Garway-Heath DF Lascaratos G Bunce C Crabb DP Russell RA Shah A. The United Kingdom Glaucoma Treatment Study: a multicenter, randomized, placebo-controlled clinical trial: design and methodology. Ophthalmology. (2013) 120:68–76. 10.1016/j.ophtha.2012.07.028 22986112

[25] Advanced Glaucoma Intervention Study,. 2. Visual Field Test Scoring Reliability. (1994). Available online at: https://pubmed.ncbi.nlm.nih.gov/7741836/ (accessed November 1, 2021). 7741836

[26] Morgan RK Feuer WJ Anderson DR. Statpac 2 glaucoma change probability. Arch Ophthalmol. (1991) 109:1690–2. 10.1001/archopht.1991.01080120074029 1841577

[27] Greaney MJ Hoffman DC Garway-Heath DF Nakla M Coleman AL Caprioli J. Comparison of optic nerve imaging methods to distinguish normal eyes from those with glaucoma. Investigative Ophthalmol Visual Sci. (2002) 43:140–5. 11773024

